# Lower serum potassium combined with lower sodium concentrations predict long-term mortality risk in hemodialysis patients

**DOI:** 10.1186/1471-2369-14-269

**Published:** 2013-12-05

**Authors:** Jyh-Chang Hwang, Ming-Yan Jiang, Charn-Ting Wang

**Affiliations:** 1 Division of Nephrology, Chi Mei Medical Center, 901 Zhonghua Rd, Yongkang Dist, Tainan 71010, Tainan, Taiwan; 2 Department of Hospital and Health Care Administration, Chia Nan University of Pharmacy and Science, Tainan, Taiwan

**Keywords:** Hypokalemia, Hyponatremia, End-stage renal disease, Malnutrition, Inflammation, Chronic kidney disease

## Abstract

**Background:**

The purpose of this study was to evaluate the combined effect of different pre-hemodialysis (HD) serum sodium (S[Na]) and potassium (S[K]) concentrations on the long-term prognosis of HD patients.

**Methods:**

A cohort of 424 maintenance HD patients (age: 58 ± 13 years, male: 47%, diabetes: 39%) from a single center were divided into four groups based on both medians of S[Na] (138.4 mmol/L) and S[K] (4.4 mmol/L): Group 1: lower S[Na] & lower S[K]: *n* = 92; Group 2: lower S[Na] & higher S[K]: *n* =113; Group 3: higher S[Na] & lower S[K]: *n* =123; Group 4: higher S[Na] & higher S[K]: *n* =96. The median observation period was 21 months.

**Result:**

By multivariate logistic regression analysis, Group 1 was characterized by hypoalbuminemia (OR = 0.37, 95%CI = 0.20-0.67), and lower normalized protein catabolism rate (nPCR) (OR = 0.37, 95% CI = 0.16-0.83). In contrast, Group 4 was characterized by higher nPCR (OR = 2.26, 95% CI = 1.05-4.86) and albumin level (OR = 2.26, 95% CI = 1.17-4.39). As compared to the reference (group 1), the HR for long-term mortality was significantly lower in Groups 3 (HR = 0.54, 95% CI = 0.34- 0.86) and 4 (HR = 0.49, 95% CI = 0.28-0.84). By multivariate Cox proportional analysis, Group 1 was an independent factor (HR = 1.74, 95% CI = 1.18-2.58) associated with long-term mortality.

**Conclusion:**

HD patients combined with lower S[K] and lower S[Na] were characterized by hypoalbuminemia, lower nPCR and a high prevalence of co-morbidity. They were associated with long-term mortality risk. On the other hand, those patients with higher levels of S[Na] and S[K] tended to have better clinical outcomes.

## Background

Hyponatremia has been reported to be associated with high mortality rates under various conditions [1-4]. Hospitalized patients with hyponatremia had an increased death risk during their hospital stay, and across 1 to 5 years’ follow-up [5]. Patients with hyponatremia were found to often be associated with more severe diseases and a high percentage of co-morbidities [6]. It has been suggested that the nature of underlying illness rather than the severity of hyponatremia better explains its associated mortality [7]. Sodium balance in hemodialysis (HD) patients is primarily dependent on two factors: dietary salt intake and sodium removal during dialysis. Salt intake during the inter-dialytic period is dependent on patient behavior and is a strong driver of volume overload, whereas in current HD practice, most sodium is removed by convection [8]. Some study has documented the impact of serum sodium concentration (S[Na]) on the long-term prognosis for HD patients [9].

Pre-HD hyperkalemia (hyperK) was associated with higher all-cause and cardiovascular mortality in HD patients [10]. The association of higher dialysate potassium concentration with increased mortality in pre-HD hyperK patients has also been observed [11]. Hypokalemia (hypoK), in contrast, is a less common concern in chronic kidney disease (CKD) patients, being a poor prognostic factor for CKD patients’ long-term survival [12,13]. In heart failure patients with CKD, a serum potassium level (S[K]) lower than 4 mmol/L was found to be associated with increased mortality and hospitalization [14].

No study has so far addressed the impact of the combined effect of pre-HD S[Na] and S[K] on long term-survival of chronic HD patients. These two markers are easily accessible and routinely monitored in clinical practice. Moreover, unexplained hypokalemia may indicate protein-energy malnutrition in dialysis patients [10], while predialysis S[Na] may be a marker of free water intake [9]. Therefore, in this study, by taking into account both of these electrolytes, we aimed to analyze the clinical features of HD patients stratified by different levels of S[Na] and S[K] and also to compare the long-term prognostic effect of each group of patients.

## Methods

This study was a cohort observational study of prospectively collected data based on the database that was constructed for outcome assurance from January 2004 to July 2008 at Chi Mei Medical Center. Excluded from the study were patients receiving HD for fewer than 6 months (n = 18), those suspected of having acute renal failure (n = 6), and those with prescribed long-term diuretics usage and potassium-reducing agents (n = 2). A total of 424 end-stage renal disease (ESRD) patients undergoing maintenance HD thrice a week, 4 hours per session, at our unit in January 2004 were enrolled in this study. Based on the median pre-dialysis S[Na] as assessed on the last mid-week dialysis session of January 2004, the patients were categorized as lower Na (S[Na] ≦ 138.4 mmol/L, n = 205) and higher Na groups (S[Na] > 138.4 mmol/L, n = 219). With median S[K] (4.4 mmol/L) as a cut-off, the patients with lower S[Na] were further subdivided into two groups: Group 1 (n = 92): patients with S[K] ≦ 4.4 mmol/L; Group 2 (n = 113): patients with S[K] > 4.4 mmol/L. Using the same criteria, the patients with higher S[Na] were also sub-divided into Group 3 (lower S[K], n = 123) and Group 4 (higher S[K], n = 96). The hollow-fiber dialyzers applied to all patients included the FB210G (Nissho Corporation, Osaka, Japan), B3-2.0 (Toray Industries, Inc, Tokyo, Japan), Hemoflow F10 HPS (Fresenius Medical Care AG, Bad, Homburn, Germany), and PSN-210 (Baxter Healthcare Corporation, McGaw Park, IL, USA). The formula of the dialysate bath used at the initiation of the study was sodium: 140.0 mmol/L, calcium: 3.0 mmol/L, potassium: 1.0 mmol/L, magnesium: 1.0 mmol/L, chloride: 104.5 mmol/L, acetate: 4.0 mmol/L, dextrose: 200 mg/dl, and bicarbonate: 35 mmol/L (Renasol® SB-1080, Renal System, Minneapolis, MN, USA). For cost reasons, from July 2004 to the end of the study we used another dialysate (Hemodialysis Concentrate A-35 & BP-11, Chi Sheng Chemical Corporation, Hsinchu, Taiwan). The formula of the second dialysate was sodium: 139.0 mmol/L, calcium: 3.0 mmol/L, potassium: 2.0 mmol/L, magnesium: 1.0 mmol/L, chloride: 106.5 mmol/L, acetate: 4.0 mmol/L, dextrose: 200 mg/dL, and bicarbonate: 39 mmol/L. No changes of potassium concentration in the dialysate bath or dialysate sodium remodeling process were prescribed though the study period.

**Figure 1:**
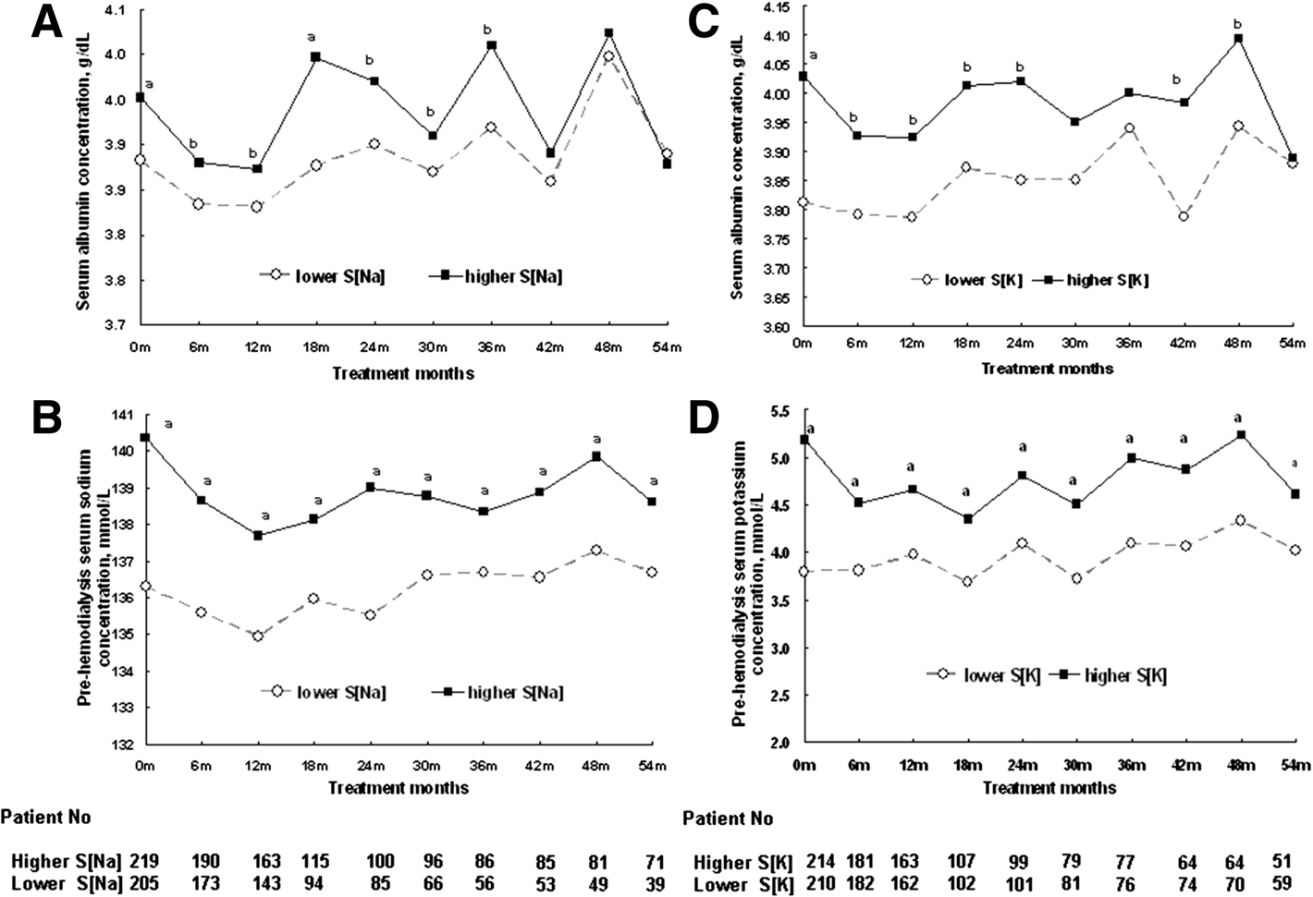
**Long-term serum sodium, potassium and albumin concentrations.** Those patients with lower serum sodium (S[Na], Groups 1 and 2) and potassium concentrations (S[K], Groups 1 and 3) at baseline had persistently lower S[Na] and S[K] **(1A and 1C)**. They also had consistently lower serum albumin concentrations **(1B and 1D)**. All the deceased patients were excluded serially. (a: p < 0.001; b: p < 0.05; compared to lower S[Na], or lower S[K], unpaired Student t tests).

Blood samples were taken in the last mid-week of January 2004 at the beginning of the study and every six months thereafter until September 2008. Pre-HD biochemical analysis including serum albumin by means of bromocresol purple, potassium, urea nitrogen (BUN), creatinine, uric acid and phosphate concentrations were measured by the Hitachi 7601–110 Automatic Analyzer (Tokyo, Japan). Serum sodium level was corrected by 1.6 mmol/L for every 100 mg/dL increase in glucose level because water transfers from the intracellular to extracellular compartment [15]. Hematocrit was measured by the Beckman Coulter LH755-A (Fullerton, CA, USA). High sensitive C-reactive protein (hs-CRP) (CardioPhase® Siemens Healthcare Diagnostics Products, GmbH, Germany) and prealbumin (immunochemical method, Dade Behring Marburg GmbH, Germany) were also checked. The data of those patients who were transferred to other centers or who died during the study were omitted on a monthly basis, as seen in Figure 1. We also evaluated the KT/V urea (Gotch formula) and normalized protein catabolism (nPCR) for all patients at the beginning of study to compare the difference in dialysis dosage and protein intake between each group [16].

The co-morbidity factors were accessed according to the past history recorded in the medical charts at the beginning of study. Coronary artery disease was defined as positive in the case of any of the following: evidence of abnormalities at coronary angiography, Tc99m-thallium scan, events of acute myocardial infarction, regional hypo- or akinesia of myocardium proved by echocardiography, and regular follow-up at the cardiovascular section for ischemic heart disease. Congestive heart failure was defined according to the criteria of New York Heart Association classification. Peripheral vascular disease was defined as chronic ulcers of the lower extremities for more than one month, and/or a history of amputation for vascular insufficiency. Stroke was defined as clinical and/or image evidence of ischemic brain syndrome or non-traumatic intracranial hemorrhage. Neoplasm was defined by the positive image and/or pathology evidence of malignancy including hematological malignancy. Chronic lung disease was defined as chronic obstructive pulmonary disease under long-term treatment with prednisolone and/or bronchodilators at the chest medicine section. Hepatitis and liver cirrhosis were evidenced by abnormal liver function for more than half a year and a positive result for chronic changes in abdominal sonography. The research was approved by the Ethics Committee of Chi Mei Medical Center and was conducted in accordance with the guiding principles for human experimentation of the Helsinki Declaration.

### Statistical analysis

Appropriate χ^2^ and ANOVA with post hoc Bonferroni tests were used for comparisons between categorical and continuous variables, respectively, between the groups, as shown in Table 1 and Figure 1. The hs-CRP comparisons among the 4 groups were undertaken with the Kruskal-Wallis one-way analysis of variance. For the evaluation of clinical features of the patients of Group 1 and Group 4, risk factors including nPCR, albumin, diabetes mellitus, gender, age at initiation of study, and HD vintage were analyzed by stepwise forward multivariate logistic regression tests (Table 2). Actuarial survival rates of the four groups categorized by different pre-HD S[Na] and S[K] were determined by the Kaplan-Meier methods, and log rank tests were employed to compare the different survival curves between groups. Cox proportional hazard methods were also performed to analyze the risk factors associated with long-term mortality (Table 3). With the exception of hs-CRP (median, inter-quartile ranges), all the other data are expressed as mean ± standard deviation. A *p* value of less than 0.05 was considered to be significant. Computations were performed with the SPSS 17.0 package for Windows (SPSS, Chicago, IL, USA).

**Table 1:** Basic demographic characteristic of the four groups of patients

	***Group1***	***Group 2***	***Group 3***	***Group 4***
	***n = 92***	***n = 113***	***n = 123***	***n = 96***
***Demographic factors***				
Diabetes mellitus, %	43	44	37	30
Male, %	40	43	50	53
Age at study, years	60 ± 13	57 ± 12	58 ± 13	57 ± 13
HD vintage, months	49.8 ± 44.6	48.9 ± 44.2	55.0 ± 45.6	48.8 ± 38.7
Ultrafiltration, L/session	2.6 ± 0.8	2.9 ± 0.7	2.5 ± 0.8	2.8 ± 0.8
nPCR, g/kg/day	1.13 ± 0.30	1.32 ± 0.34^d^	1.21 ± 0.34^e^	1.33 ± 0.28^d^
Kt/V^*^	1.45 ± 0.25	1.43 ± 0.26	1.40 ± 0.25	1.40 ± 0.23
Mean pre-HD blood pressure, mmHg	95 ± 15	100 ± 14	94 ± 16	97 ± 15
ACEI and/or ARB, %	28	28	28	21
Karnofsky score	72 ± 18	79 ± 16^c^	76 ± 18	80 ± 16^c^
***Clinical comorbidity, %***				
Coronary artery disease	27	17	13	20
Congestive heart failure	2	5	2	3
Peripheral vascular disease	8	11	4	3
Stroke	14	12	12	7
Neoplasm	8	7	12	3
Chronic lung disease	2	0	1	1
Liver cirrhosis and/or hepatoma	5	1	4	3
No co-morbidity	47	64^a^	58	67^a^
Death rate, %(n)	40 (37)	28 (32)	28 (35)	23 (22)^a^
***Laboratory data***				
hs-CRP, mg/L, median	16.32	7.69	8.24	3.78
(1st -3rd quartile ranges)	(2.05-20.4)	(0.70-8.73)^g^	(0.88-7.33)^g^	(0.93-4.85)^h^
Pre-albumin, mg/dL	29.1 ± 10.0	33.6 ± 9.3^c^	32.6 ± 9.4^c,e^	36.2 ± 8.9^d^
Albumin, g/dL	3.7 ± 0.5	4.0 ± 0.4^d^	3.9 ± 0.4^e^	4.1 ± 0.4^d^
Sodium, mmol/L	134.6 ± 2.9	135.4 ± 2.3^f^	139.6 ± 2.1^d^	139.3 ± 2.0^d^
Potassium, mmol/L	3.8 ± 0.5	5.3 ± 0.7^d,e^	3.8 ± 0.5^f^	5.0 ± 0.5^d^
Phosphate, mg/dL	4.2 ± 1.5	5.2 ± 1.7^d^	4.4 ± 1.4^f^	5.2 ± 1.4^d^
BUN, mg/dL	65 ± 23	85 ± 24^d^	67 ± 20^f^	80 ± 17^d^
Creatinine, mg/dL	8.5 ± 2.8	10.0 ± 2.5^d^	9.8 ± 2.5^c^	10.6 ± 2.5^d^
Uric acid, mg/dL	7.2 ± 1.5	8.2 ± 1.8^d^	7.5 ± 1.5^e^	8.1 ± 1.2^d^
Hematocrit, %	27.0 ± 5.3	28.2 ± 5.1	26.8 ± 4.3	27.9 ± 4.1

*Abbreviations*: *HD* hemodialysis, *nPCR* normalized protein catabolism rate, *ACEI* angiotensinogen converting enzyme inhibitor, *ARB* angiotensin receptor blockade, *hs-CRP* high sensitivity C-reactive protein, *BUN* blood urea nitrogen. *: Gotch formula.

Statistics:

a: p < 0.05 vs. group 1 (χ2 tests).

c: p < 0.05, d: p < 0.001 vs. group 1 (ANOVA with post hoc Bonferroni tests).

e: p < 0.05, f: p < 0.001 vs. group 4 (ANOVA with post hoc Bonferroni tests).

g: p < 0.05, h: p < 0.001 vs. group 1(Kruskal–Wallis one-way analysis of variance).

**Table 2:** Multivariate logistic regression analyses of the clinical associations of Groups 1 and 4

		**95% CI**		
	**OR**	**Lower**	**Upper**	**p**
***A. Group 1***^***a***^				
nPCR, g/kg/day	0.37	0.16	0.83	0.02*
Albumin, g/dL	0.37	0.20	0.67	<0.001*
***B. Group 4***^***b***^				
nPCR, g/kg/day	2.26	1.05	4.86	0.04*
Albumin, g/dL	2.26	1.17	4.39	0.02*

a. dependent variable: Group 1 (n = 92) vs. others (Groups 2,3, and 4; n = 332).

b. dependent variable: Group 4 (n = 96) vs. others (Groups 1,2, and 3; n = 328).

*Abbreviations*: *CI* confidence interval, *nPCR* normalized protein catabolic rate, *HD* hemodialysis.

*Adjusted for diabetes mellitus, gender, age at initiation of study, and HD vintage.

**Table 3:** Cox proportional method for evaluation the risk factors related to long-term mortality

		**Univariate**				**Multivariate**		
	**HR**	**95% CI**		**p**	**HR**	**95% CI**		**p**
Group 1 (vs. other 3 groups)*	1.78	1.21	2.61	0.003	1.74	1.18	2.58	0.006^#^
Sodium, mmol/dL	0.92	0.88	0.97	0.002				
Potassium, mmol/dL	0.80	0.65	0.98	0.03				
Diabetes (vs. non-diabetes)	1.64	1.15	2.34	0.006	1.71	1.17	2.49	0.005
Male (vs. female)	1.06	0.75	1.51	0.74	1.35	0.95	1.93	0.10
Age^※^, year	1.05	1.04	1.07	<0.001	1.05	1.04	1.07	<0.001
HD duration, month	1.00	1.00	1.01	0.26	1.00	0.99	1.01	0.10

*Abbreviations*: *CI* confidence interval, *HD* hemodialysis, *: group 1 vs. the other 3 groups, death rate: 37/92 (40%) vs. 89/332 (27%), ※: Age at the initiation of study.

#: adjusted for diabetes mellitus, gender, age, and HD vintage.

## Results

### Basic demographic characteristics of four groups of patients

Although the differences between Groups 1 and 3 were not statistically significant, Group 1 had lower nPCR, Karnofsky score, and percentage of non-comorbidity than Groups 2 and 4 (Table 1). Serum levels of albumin, phosphate, uric acid, and pre-HD BUN were also significantly lower in Group 1compared to Groups 2 and 4. Among the 4 groups, Group 1 had the highest hs-CRP and lowest pre-albumin and Cr levels. On the other hand, Group 4 had statistically higher nPCR, prealbumin, albumin, phosphate, BUN, and uric acid levels than those of Group 3. There were no significant differences in gender ratio, age at the initiation of study, HD vintage, Kt/V, ultrafiltration rate, mean blood pressure, percentage for DM, use of ACEI and/or ARB and hematocrit levels among the four groups of patients.

In a comparison of each electrolyte, the lower S[K] group (Groups 1 and 3) had significantly higher hs-CRP level (3.85 mg/dL, 1.13-11.7 mg/dL vs. 2.65 mg/dL, 0.78-6.64 mg/dL, p = 0.015), lower nPCR (1.18 ± 0.33 vs. 1.32 ± 0.31 g/kg/day, p < 0.001) and serum albumin concentration (3.8 ± 0.5 vs. 4.0 ± 0.4 g/dL, p < 0.001) compared to the their high group counterparts. On the other hand, higher hs-CRP level (4.32 mg/L, 1.02-11.8 mg/L vs. 2.31 mg/dL, 0.92-5.81 mg/L, p = 0.002), percentage of ultrafiltration rate to dry weight (5.1 ± 1.6% vs. 4.6 ± 1.4%, p = 0.001) and BUN-to-creatinine ratio (8.50 ± 3.27 vs. 7.39 ± 2.08, p < 0.001) were found in the lower S[Na] group (Groups 1 and 2) compared to the high S[Na] group (Groups 3 and 4).

### Comparison of the long-term survival divided by serum sodium and potassium concentrations

**Figure 2:**
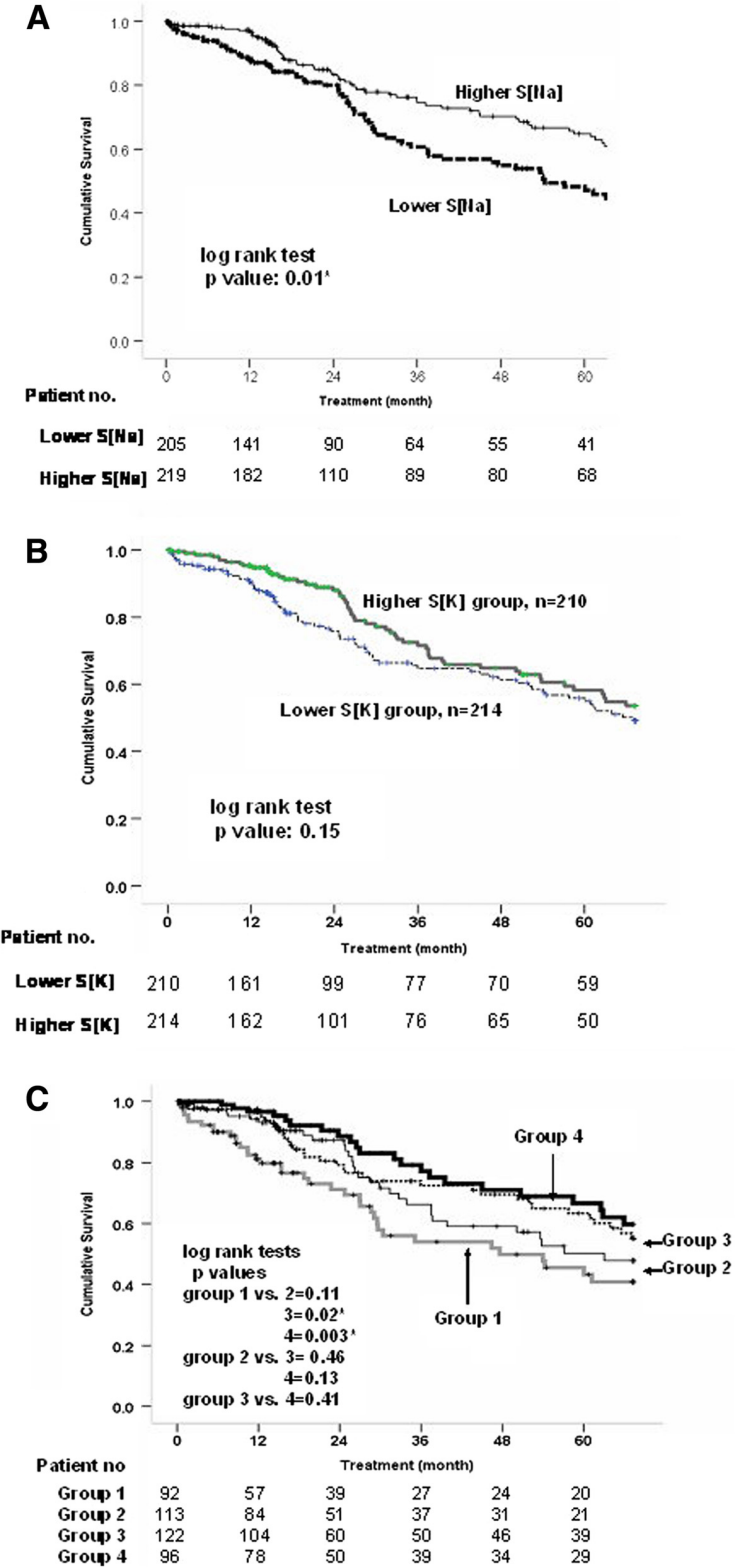
**Comparison of the long-term survival divided by serum sodium and potassium concentrations.** The patients with low serum sodium concentration (S[Na], Groups 1, and 2) had a poorer cumulative survival than their counterparts (p = 0.010, **2A**). Cumulative survival rate of lower serum potassium (S[K]) group was non-significantly lower than that of the higher S[K] group (p = 0.147, **2B**). After investigating the combination of the two electrolytes, Group 1 was found to have a poorer outcome compared to Groups 3 and 4. There was no difference among Groups 2, 3, and 4 **(2C)**.

**Figure 3:**
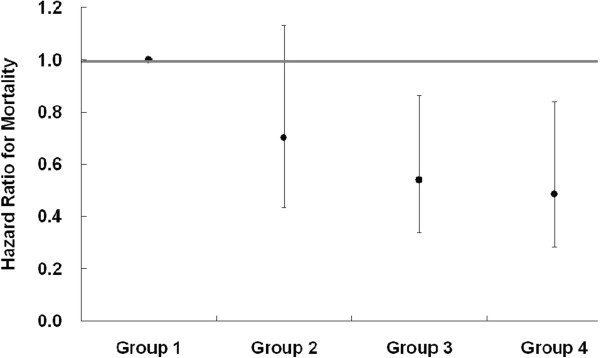
**Hazard ratios for mortality among each group after adjusted risk factors.** After adjustment for DM, gender, HD duration, and age at the beginning of study, the hazard ratio for mortality was significantly lower in Group 3 (HR = 0.54, 95% CI = 0.34-0.86), followed by Group 4 (HR = 0.49, 95% CI = 0.28-0.84).

Lower S[Na] patients had a significantly poorer cumulative survival than the higher S[Na] counterparts (p = 0.01) (Figure 2A). The cumulative survival rate of the lower S[K] group was non-significantly lower than the higher S[K] counterparts (p = 0.15) (Figure 2B). In the combined analysis of the two electrolytes, Group 1 had a poorer outcome compared to Groups 3 and 4, but there was no statistical difference among Groups 2, 3, and 4 (Figure 2C). After adjustment for DM, gender, HD duration, and age at the beginning of study, as compared to the reference (Group 1), the hazard ratio for mortality was significantly lower in Groups 3 (HR = 0.54, 95% CI = 0.34-0.86) and 4 (HR = 0.49, 95% CI = 0.28-0.84) (Figure 3).

### Characteristics of patients with Groups 1 and 4

As shown in Figure 2, those patients with both lower S[Na] and S[K] (Group 1) had the worst long-term prognosis while those patients in Group 4, with both higher S[Na] and S[k], had the better outcomes. Therefore, we attempted to characterize the clinical association of these two groups. After adjustment for DM, gender difference, age at initiation of study, and HD vintage in multivariate logistic regression tests, Group 1 was associated with lower nPCR (OR: 0.37; 95% CI = 0.16-0.83) and hypoalbuminemia (OR: 0.37; 95% CI = 0.20-0.67). Conversely, Group 4 was associated with higher levels of nPCR (OR: 2.26; 95% CI = 1.05-4.86) and albumin (OR: 2.26; 95% CI = 1.17-4.39).

### Long-term serum sodium, potassium and albumin concentration changes

Figure 1 shows the serial changes of S[Na] and S[K] throughout the study period. Those patients with either lower S[Na] or S[K] at baseline had persistently lower levels of S[Na] and S[K] (Figure 1B & D). Both also had consistently lower serum albumin concentrations (Figure 1A & B).

### Cox proportional method for evaluation the risk factors related to mortality

As can be seen in Table 3, Group 1, lower S[Na], lower S[K], DM, and old age were risk for death in univariate Cox proportional analysis. After adjusting for DM, gender, age at the initiation of study, and HD vintage by multivariate study, Group 1 was found to be an independent factor for long-term mortality (HR: 1.74; 95% CI: 1.18-2.58).

## Discussion

This is the first report to address the combination effect of S[Na] and S[K] on the long-term survival of chronic HD patients. The main finding was that patients with lower S[K] superimposed with lower S[Na] were characterized by hypoalbuminemia and lower nPCR level. They were also associated with higher hs-CRP level, and more co-morbidity. The combination of lower levels of both electrolytes in HD patients was proven to be an independent factor leading to a high long-term mortality. In contrast, HD patients with higher levels of both electrolytes tended to have better outcomes.

Recently, lower pre-HD S[Na] has been reportedly associated with an increased risk of all-cause mortality in HD patients [9,17], but the mechanism for this remains obscure. We suggest several hypotheses. A high catabolic rate and inflammation status may be a cause, since we found that patients with lower S[Na] had high levels of hs-CRP and BUN-to-creatinine ratio (BUN/Cr) in this study. A high ratio of BUN/Cr has been recognized as a marker of high catabolism rate in clinical practice. Hypervolemia may be another factor. The patients with lower S[Na] had a significantly higher level of ultrafiltration rate. This may have been caused by either previous inadequate ultrafiltration and/or over-ingestion of fluid during the inter-dialysis period, eventually leading to chronic fluid retention. Higher interdialytic weight gain was also correlated with poor survival and increased cardiovascular death [18], with patients of lower S[Na] as compared to their higher S[Na] counterparts also having a higher percentage of DM. Diabetes patients with impaired autonomic nervous system tended to develop intra-dialysis hypotension [19], resulting in an inadequate ultrafiltration rate and a failure to achieve a real dry weight. Both these factors lead to chronic fluid retention and lowering of the S[Na]. Hyperglycemia, dipsogenic factors, and poor compliance to salt restriction with resultant high fluid intake might also be contributory factors to lower S[Na] [20]. Eventually, all of these factors were associated with hypervolemia, thus resulting in congestive heart failure and mortality risk. Hypervolemia is an important factor contribution to the long-term mortality of HD patients [21].

HyperK in HD patients was demonstrated to have a high risk of mortality by Kovesdy. However, we found that the patients with lower S[K] were associated with non-significantly worse long-term outcomes. HypoK patients with S[K] less than 3.5 mmol/L were reported to have a higher mortality risk than patients with higher S[K] [12]. This observational discrepancy may result from different patient characteristics. Our patients, for example, tended to suffer more frequently from malnutrition than metabolic syndrome. BMI values of our patients were significantly lower (21–22 vs. 26) [12]. Some of their patients were dialyzed with more than 2–3 mmol/L in dialysate K bath [12]. CKD patients not on dialysis with low or even low-normal S[K] were observed to be at a higher risk of dying than those with mild to moderate hyperkalemia. In our study, lower S[K] patients were associated not only with lower levels of nPCR and albumin, but also with a high level of hs-CRP. These features signify that these patients are characterized with more severe malnutrition and higher inflammation status. We hypothesize that HD patients with lower S[K] may also be under a catabolic rate and protein-energy wasting status [22]. They also had a higher prevalence of co-morbid conditions, thus further associating lower S[K] with high mortality.

Group 1 was associated with lower levels of serum albumin and nPCR. This could explain why “Group 1” lost statistical significance in predicting long-term outcome in model 2 Cox proportional analyses after the addition of “albumin” as a covariate. In clinical practice, HD patients having low levels of both serum albumin and nPCR are invariably indicative of the worst clinical conditions [23]. Both hypoalbuminemia and low nPCR are also indicated as good surrogate markers for all-cause mortality in HD patients. This could explain why the patients in Group 1 were associated with worse long-term prognoses.

From our data, lower S[Na] is definitely much worse than higher S[Na], and lower S[K] is tends to be a little worse than higher S[K]. When combining the two, the long-term effects of lower S[K] are a cause for concern, but they may be overlooked. While extreme hyperkalemia may always be serious, when cutting in the middle, patients are better off being on the relatively higher S[K] side rather than the lower S[K] side.

In this study, we found consistency of S[Na] and S[K] in the chronic HD patients over the course of 54 months. This invariable phenomenon has been reported before [12,24]. Hypoalbuminemia was persistently noted in the patients characterized by either lower S[Na] or lower S[K] throughout the whole study period. This stability in low serum albumin concentrations suggests that those patients with a lower level of either electrolyte share some intrinsic factors, which cause long-standing malnutrition and sub-clinical inflammation. This could explain the high mortality rate in these patients.

Contrary to Group 1, Group 4 had the lower prevalence of diabetes and level of hs-CRP, as well as higher levels of serum pre-albumin, albumin, creatinine, and nPCR. This means that these patients are under relatively better nutrition and suffer rather less inflammation. Group 4 patients also had a higher Karnofsky score than Group 1. These features could explain the better survival in these patients compared to the other groups.

As far as we know, no data has yet been reported on the impact of the combined effect of S[K] and S[Na] on the long-term prognosis in chronic HD patients. One strength of our study is the 4.5-year length of follow-up. But there are also some limitations to this study. First, we did not check body volume directly, since we suspected that lower S[Na] was the result of hypervolemia. Second, the analysis was based on single sodium and potassium values. Moreover, the dialysate composition was changed shortly after baseline, which also may have influenced the results. However, these omissions are not expected to distort the deleterious impact of lower S[K] superimposed with lower S[Na] on the long-term prognosis of HD patients.

## Conclusions

In conclusion, our data demonstrated that HD patients with lower levels of both S[K] and S[Na] were associated with poor cumulative survival. They were associated with hypoalbuminemia, and lower nPCR levels. Independent of the traditional risk factors including DM, age, gender and HD vintage, both electrolytes under lower levels are able to predict the worst long-term outcomes for HD patients. On the other hand, those patients with higher levels of both S[Na] and S[K] were found to have better long-term prognoses. Although we suspect that persistent malnutrition, high inflammation status, more co-morbidity risks, and chronic hypervolemia in patients with lower S[Na] and S[K] were the main causes leading to higher mortality, the real mechanism still needs further clarification.

## Consent

The research ethics committee of the Chi Mei Medical Center has approved this study (IRB no. 10109–012). Written informed consent was obtained from the patient for the publication of this report and any accompanying images.

## Competing interests

The authors declare that they have no competing interests.

## Authors’ contributions

JC designed the research; JC and MY reviewed the references; JC and CT analyzed the data, and JC wrote the paper. All authors read and approved the final manuscript.

## Pre-publication history

The pre-publication history for this paper can be accessed here:

http://www.biomedcentral.com/1471-2369/14/269/prepub

## References

[B1] Kim WR Biggins SW Kremers WK Wiesner RH Kamath PS Benson JT Edwards E Therneau TM Hyponatremia and mortality among patients on the liver-transplant waiting list N Engl J Med 2008 14 1018 1026 10.1056/NEJMoa0801209 18768945 PMC4374557

[B2] Boin IF Capel C Jr Ataide EC Cardoso AR Caruy CA Stucchi RS Pretransplant hyponatremia could be associated with a poor prognosis after liver transplantation Transplant Proc 2010 14 4119 4122 10.1016/j.transproceed.2010.10.019 21168641

[B3] Shorr AF Tabak YP Johannes RS Gupta V Saltzberg MT Costanzo MR Burden of sodium abnormalities in patients hospitalized for heart failure Congest Heart Fail 2011 14 1 7 10.1111/j.1751-7133.2010.00206.x 21272220

[B4] Nair V Niederman MS Masani N Fishbane S Hyponatremia in community- acquired pneumonia Am J Nephrol 2007 14 184 190 10.1159/000100866 17356253

[B5] Waikar SS Mount DB Curhan GC Mortality after hospitalization with mild, moderate, and severe hyponatremia Am J Med 2009 14 857 865 10.1016/j.amjmed.2009.01.027 19699382 PMC3033702

[B6] Wald R Jaber BL Price LL Upadhyay A Madias NE Impact of hospital- associated hyponatremia on selected outcomes Arch Intern Med 2010 14 294 302 10.1001/archinternmed.2009.513 20142578

[B7] Chawla A Sterns RH Nigwekar SU Cappuccio JD Mortality and serum sodium: do patients die from or with hyponatremia? Clin J Am Soc Nephrol 2011 14 960 965 10.2215/CJN.10101110 21441132 PMC3087791

[B8] Santos SF Peixoto AJ Sodium balance in maintenance hemodialysis Semin Dial 2010 14 549 555 10.1111/j.1525-139X.2010.00794.x 21175831

[B9] Waikar SS Curhan GC Brunelli SM Mortality associated with low serum sodium concentration in maintenance hemodialysis Am J Med 2011 14 77 84 10.1016/j.amjmed.2010.07.029 21187188 PMC3040578

[B10] Kovesdy CP Regidor DL Mehrotra R Jing J McAllister CJ Greenland S Kopple JD Kalantar-Zadeh K Serum and dialysate potassium concentrations and survival in hemodialysis patients Clin J Am Soc Nephrol 2007 14 999 1007 10.2215/CJN.04451206 17702709

[B11] Al-Ghamdi G Hemmelgarn B Klarenbach S Manns B Wiebe N Tonelli M Dialysate potassium and risk of death in chronic hemodialysis patients J Nephrol 2010 14 33 40 20091484

[B12] Hwang JC Wang CT Chen CA Chen HC Hypokalemia is associated with increased mortality rate in chronic hemodialysis patients Blood Purif 2011 14 254 261 10.1159/000325226 21849775

[B13] Korgaonkar S Tilea A Gillespie BW Kiser M Eisele G Finkelstein F Kotanko P Pitt B Saran R Serum potassium and outcomes in CKD: insights from the RRI-CKD cohort study Clin J Am Soc Nephrol 2010 14 762 769 10.2215/CJN.05850809 20203167 PMC2863985

[B14] Bowling CB Pitt B Ahmed MI Aban IB Sanders PW Mujib M Campbell RC Love TE Aronow WS Allman RM Bakris GL Ahmed A Hypokalemia and outcomes in patients with chronic heart failure and chronic kidney disease: findings from propensity-matched studies Circ Heart Fail 2010 14 253 260 10.1161/CIRCHEARTFAILURE.109.899526 20103777 PMC2909749

[B15] Katz MA Hyperglycemia-induced hyponatremia - calculation of expected serum sodium depression N Engl J Med 1973 14 843 844 10.1056/NEJM197310182891607 4763428

[B16] Depner TA Daugirdas JT Equations for normalized protein catabolic rate based on two-point modeling of hemodialysis urea kinetics J Am Soc Nephrol 1996 14 780 785 8738814 10.1681/ASN.V75780

[B17] Hecking M Karaboyas A Saran R Sen A Hörl WH Pisoni RL Robinson BM Sunder-Plassmann G Port FK Predialysis serum sodium level, dialysate sodium, and mortality in maintenance hemodialysis patients: the dialysis outcomes and practice patterns study (DOPPS) Am J Kidney Dis 2012 14 238 248 10.1053/j.ajkd.2011.07.013 21944663

[B18] Kalantar-Zadeh K Regidor DL Kovesdy CP Van Wyck D Bunnapradist S Horwich TB Fonarow GC Fluid retention is associated with cardiovascular mortality in patients undergoing long-term hemodialysis Circulation 2009 14 671 679 10.1161/CIRCULATIONAHA.108.807362 19171851 PMC2773290

[B19] Wu JS Lu FH Yang YC Chang CJ Postural hypotension and postural dizziness in patients with non-insulin-dependent diabetes Arch Intern Med 1999 14 1350 1356 10.1001/archinte.159.12.1350 10386511

[B20] Gary Abuelo J Large interdialytic weight gains: causes, consequences, and corrective measures Semin Dial 1998 14 25 32

[B21] Chazot C Wabel P Chamney P Moissl U Wieskotten S Wizemann V Importance of normohydration for the long-term survival of haemodialysis patients Nephrol Dial Transplant [Epub ahead of print] 10.1093/ndt/gfr678 22253067

[B22] Shinaberger CS Kilpatrick RD Regidor DL McAllister CJ Greenland S Kopple JD Kalantar-Zadeh K Longitudinal associations between dietary protein intake and survival in hemodialysis patients Am J Kidney Dis 2006 14 37 49 10.1053/j.ajkd.2006.03.049 16797385

[B23] Block GA Klassen PS Lazarus JM Ofsthun N Lowrie EG Chertow GM Mineral metabolism, mortality, and morbidity in maintenance hemodialysis J Am Soc Nephrol 2004 14 2208 2218 10.1097/01.ASN.0000133041.27682.A2 15284307

[B24] Peixoto AJ Gowda N Parikh CR Santos SF Long-term stability of serum sodium in hemodialysis patients Blood Purif 2010 14 264 267 10.1159/000274460 20068291

